# Demonstration of two novel methods for predicting functional siRNA efficiency

**DOI:** 10.1186/1471-2105-7-271

**Published:** 2006-05-29

**Authors:** Peilin Jia, Tieliu Shi, Yudong Cai, Yixue Li

**Affiliations:** 1 Bioinformatics Center, Shanghai Institutes for Biological Sciences, The Chinese Academy of Sciences, 320 Yueyang Road, Shanghai 200031, China; 2 Graduate School of the Chinese Academy of Sciences, 19 Yuquan Road, Beijing 100039, China; 3 Shanghai Center for Bioinformation Technology, 100 Qinzhou Road, Shanghai 200235, China; 4 CAS-MPG Partner Institute for computational biology, Shanghai, Institute of Biological Scienices, Chinese Academy of Sciences, 320 Yue Yang Road, Shanghai, China; 5 Department. of Biomolecular Sciences, UMIST, Manchester M60 1QD, UK; 6 Life Science School. of Shanghai Jiao Tong University, Shanghai, China

## Abstract

**Background:**

siRNAs are small RNAs that serve as sequence determinants during the gene silencing process called RNA interference (RNAi). It is well know that siRNA efficiency is crucial in the RNAi pathway, and the siRNA efficiency for targeting different sites of a specific gene varies greatly. Therefore, there is high demand for reliable siRNAs prediction tools and for the design methods able to pick up high silencing potential siRNAs.

**Results:**

In this paper, two systems have been established for the prediction of functional siRNAs: (1) a statistical model based on sequence information and (2) a machine learning model based on three features of siRNA sequences, namely binary description, thermodynamic profile and nucleotide composition. Both of the two methods show high performance on the two datasets we have constructed for training the model.

**Conclusion:**

Both of the two methods studied in this paper emphasize the importance of sequence information for the prediction of functional siRNAs. The way of denoting a bio-sequence by binary system in mathematical language might be helpful in other analysis work associated with fixed-length bio-sequence.

## Background

RNA interference (RNAi) is a biological mechanism by which double stranded molecules inhibit gene expression by mediating sequence-specific mRNA degradation [1]. The process starts when dsRNA molecules are degraded into short interfering RNA (siRNA) molecules, about 21–23 nucleotides in length, by the RNase enzyme Dicer. These siRNAs are subsequently incorporated into a silencing complex called RISC (RNA-induced silencing complex), which identifies and destroys complementary RNAs [2]. RNAi is an evolutionally conserved mechanism for targeted repression of gene expression that has been developed into an experimental tool for silencing specific genes across systems [1,2]. Gene silencing efficiency has varied greatly among siRNAs targeting at different positions of a specific gene or at different genes. Therefore, efficient predictive tools are in high demand for siRNA design.

Many efforts have been made trying to develop computational methods which can provide improved prediction of potentially functional siRNA [2-6]. These methods have been based on various parameters including sequence features, energy features, RNA secondary structure features, and so on [7]. Each of these characteristics may affect RNAi efficiency. Scoring algorithms have been widely utilized since statistically significant nucleotide base preferences can easily be applied in the construction of scoring algorithms [4]. Data mining methods have also been employed in siRNA prediction and shown promising performance [8].

In the field of machine learning, siRNA prediction can be considered as a typical pattern recognition or classification problem. Based on this consideration we developed two kinds of algorithms, both of which achieved high performence. The first one is a sequence based statistical model that has been successfully used in signal peptide prediction [9]. In this paper, we applied this method to siRNA analysis and also obtained satisfactory prediction quality. In addition, we employed Vapnik's Support Vector Machine (SVM) as an alternative solution to this problem [10]. SVM has many attractive characteristics, including over fitting avoidance, large feature spaces handling and key information extracting from a given data set. This approach has provided satisfactory performance for a wide variety of classification problems in bioinformatics areas including microarray data analysis [11], protein structure classification [12], signal peptide prediction [13] and protein subcellular localization identification [14] and so on. SVM has already been applied to predict the efficacy of short oligonucleotides in antisense and siRNAs by Satron and got good results [8,15]. Unlike above methods, we use the SVM algorithm in a novel way by introducing a binary system to denote sequences of fixed-length. Besides the binary system, thermodynamic profile and nucleotide composition were also introduced to construct the vector space of SVM.

## Results

As we have known, to objectively assess a prediction method, a homogeneous and sufficiently large dataset is of high importance. It should also be very careful to combine datasets from different resources, because the efficiency of a siRNA changes variously under different biological and experimental conditions. Fortunately, the recently published Dieter's dataset from a high-throughput assay makes it possible to break the bottleneck of directly comparing different source datasets [16]. Besides, in order to compare with the former work, we also use the dataset from Satron, which combines published siRNAs from several researches and has been used in Satron's research [8]. To guarantee the unification of the dataset and to make the training processes of our algorithms objectively, we trained those two datasets separately and took the results from Dieter's dataset as the main evaluation measurement. Moreover, the Satron's dataset provides siRNAs with 19 nt in length while the Dieter's dataset with 21 nt in length, which also makes it impossible to train them together.

Whether a siRNA can be denoted as functional or non-functional depends on its ability of silencing a target gene, which is often measured by the value of siRNA inhibitory activity. Thus we generated the positive and negative subsets according to the level of inhibitory activity. During our research, we took three cut-off values, 0.5, 0.6 and 0.7 to generate six combinations of positive and negative subsets for the two datasets (table 1). That is, siRNAs in the positive dataset have their inhibitory activity greater than the cutoff value and siRNAs in the negative dataset have their inhibitory activity less then the cutoff value.

For each of the six combinations, both of the self-consistency and the jackknife test were performed. Especially, during the training processes of SVM algorithm, to compare the contributions of each of the three attributes, all seven combinations have been performed independently, which are "binary, thermodynamic and composition", "binary and thermodynamic", "thermodynamic and composition", "binary and composition", "binary only", "thermodynamic only", "composition only". All the results from the two methods have been listed from table 2 to table 4 (for detailed results please see additional file 2 and 3).

Those results in table 2, 3 and 4 clearly show that both of the methods got high performance on Dieter's dataset. During the process of jackknife test, as for the sequence-based statistical model, the accuracy on Dieter's dataset are 89.35%, 89.47% and 89.43% for cut-off value 0.7, 0.6 and 0.5, while on Satron's dataset are 70.94%, 70.59%, 70.23% for cut-off value 0.7, 0.6 and 0.5 (table 2). Also the process of jackknife test, as for SVM, when all of the three attributes are used, the accuracy on Dieter's dataset achieved 94.78%, 94.65% and 94.65% for cut-off value 0.7, 0.6 and 0.5, respectively (table 3), while on Satron's dataset the accuracy are 78.07%, 71.66% and 72.55% for cut-off value 0.7, 0.6 and 0.5 (table 4). Obviously, the method of SVM performed better than the sequence-based statistical model, and both of the two methods performed better on Dieter's dataset than Satron's dataset. For the three kinds of attributes, namely binary, thermodynamic and composition, the highest accuracy achieved when only the binary attribute is used for SVM training processes against Dieter's dataset during the jackknife test with any of the cut-off value. For Satron's dataset, the highest accuracy appeared when "binary and composition", or "thermodynamic and composition", or "binary, composition and thermodynamic" are used during the jackknife process.

From table 3 and table 4 we can also see that the values of sensitivity and specificity differ from each other greatly during SVM training processes tested on Satron's dataset with cut-off value of 0.5, 0.6 or 0.7 and on Dieter's dataset with cut-off value of 0.5 or 0.7. For example, for Satron's dataset when the cut-off value 0.7 was used, we got a positive subset containing 141 siRNAs and a negative subset containing 420 siRNAs. We use these datasets as the input for jackknife test by SVM and got the sensitivity of 21.99% and the specificity of 96.09%. For the six combinations listed in table 1, only the one generated by Dieter's dataset using cut-off value of 0.6 has no this problem.

To compare with Dieter's work [16], we constructed 15 sub-datasets according to Dieter's description to perform the training and testing test by the sequence-based model and SVM model we have constructed in this work. Each of the cut-off value of 0.5, 0.6 or 0.7 was performed separately. Table 5 lists the results of the two methods with cut-off value of 0.6. The other results are detailed in supplemental file (see additional file 4). From table 5, we can see that both of the sequence-based model and SVM methods show great improvement than Dieter's work. The highest pearson correlation coefficient reaches 0.9771 by SVM and 0.8562 by sequence-based model when the training dataset is "All (2182)" and the testing dataset is "All (249)", while the corresponding coefficient by Dieter et al is 0.66. Whatever the cut-off value is, both of the two methods got high correlation coefficient, especially the SVM method.

## Discussion

### Comparing the three attributes in the vector space for SVM training

During the training processes executed by SVM, we constructed three kinds of attributes which are the binary representation, the thermodynamic profile and the nucleotide composition of the sequence. All the seven combinations of the three attributes have been chosen as the input of SVM training machine to find their contributions and all the results are listed in supplemental file (see additional file 3). From the results in table 3 and table 4, we can definitely come to the conclusion that the binary representation system plays the most important role. For both of the datasets, the accuracy of the prediction will be improved greatly whenever the binary system has been added. Take Dieter's dataset for example, the four attribute combinations, all of which contain the binary system, have their accuracy higher than 90%, with about 10~25% improvement comparing with that of the rest three combinations. Neither the thermodynamic profile nor the nucleotide composition can provide such an obvious enhancement. We refer this phenomenon to the fact that the binary representation, though not indicate any biological or chemistry property of the sequence, might carry the sequence speciality such as sequence order, base preferences at certain sites, etc. Previous studies have proved that effective siRNAs show base preferences at positions 3, 10, 13 and 19 of the sense strand [4]. Other sequence characteristics have been also noted by Kumiko Ui-Tei *et al *[5]. The high correlation coefficients in our research emphasized the fact that the sequence of a potential siRNA oligo is intimately correlated with its function.

Actually, what we discussed here is ubiquitous in the field of bio-sequence based function prediction – the problem is how to describe a bio-sequence in a suitable mathematical language. The binary system performs well in this problem of siRNA prediction. It also works well in other machine learning areas in bioinformatics, such as prediction work about protein signal sequences and their cleavage sites [13]. This kind of binary system can be used to represent qualitative concepts such as season, blood group, etc. Generally speaking, when a variable has n types, the binary system use n-dimension vector to denote each one of the n type, with the value of the *i*th dimension equals 1 and all other dimensions equals 0 for the *i*th type. We suggest this binary model might be used in other sequence based prediction works.

The nucleotide composition, including single nucleotide and di-nucleotide compositions, indicating sequence profile at a certain level thus also has its special role in training process. Also take Dieter's dataset for example, when the input attributes are "Thermodynamic and Composition", "Thermodynamic only" or "Composition only", the accuracy are 85.97%, 78.69% or 81.65% respectively for cut-off value as 0.7, 83.83%, 76.31% or 80.09% for 0.6, and 83.67%, 77.46% or 79.47% for 0.5 (see table 3). Briefly speaking, the attribute of composition profile provides about 6~7% enhancement for the prediction, without considering the possibly weakening or enhancing interaction between two attributes. However, it should be noted that composition profile is not sufficient enough since two oligos having the same nucleotide composition might differ greatly in sequence order. Thus we proposed to use this attribute together with other attributes to provide enough information for mapping a siRNA oligo onto the vector space.

As has been proved by many experimental researches, the thermodynamic profile of a siRNA plays important role in the RNA interference mechanism [17]. That is why we take the thermodynamic profile as the input of SVM machine. The results from our work are consistent with the previous work. When the "thermodynamic only" attribute was provided as the input for SVM training processes, the accuracy during jackknife test achieved 74.87%, 68.27% and 63.99% for Straon's dataset with cut-off value as 0.7, 0.6 and 0.5, while the corresponding value are 78.69%, 76.31% and 77.46% for Dieter's dataset. This sufficiently showed the importance of the thermodynamic character during the RNA interference process. However, considering all the seven combinations of the three attributes, we suggest it is better to put them together as the input of SVM machine.

To avoid redundancy between the three attributes, we calculated the Pearson correlation coefficiency. For Dieter's dataset, the correlation coefficiency between the nucleotide composition and the thermodynamic profile is 1.381E-4, the nucleotide composition and binary system is 1.132E-5, and the thermodynamic profile and binary system is 3.092E-4. The low correlation between these attributes indicates that it is proper to combine them together for prediction.

### Balancing the biased dataset in SVM training

From table 3 and table 4, we can see that when the five subsets, which are Satron's dataset with cut-off value of 0.5, 0.6 and 0.7, and Dieter's dataset with cut-off value of 0.5 and 0.7, are taken as the input datasets for SVM training, the sensitivity and specificity apart from each other abnormally. On the one hand, this disparity between sensitivity and specificity appears to be much greater when the number of records in positive dataset departs further from the number in the negative dataset or when the dimension of the vector space turns lower. For example, the value of sensitivity and specificity present the greatest disparity with 0.00% and 100.00% respectively under the following conditions: the vector space are constructed with only the thermodynamic or composition attribute, and the dataset is that from Straon's data with cut-off value as 0.7, in which the number of records in the positive dataset is almost three times of the number in the negative dataset. Even when the vector space is expanded by all of the three attributes, and we got the smallest difference in the record number between the positive and negative datasets, this disparity is more than 20% (see table 5, vector space expanded by the attributes "binary, thermodynamic and composition", training set as from Satron's dataset with cut-off value of 0.5, during jackknife test). On the other hand, the disparity is not obvious for some certain combinations of attributes when the dataset is large enough. This can be seen from Dieter's datasets with cut-off value of 0.5 or 0.7 in the vector space of "binary, thermodynamic and composition", "binary and thermodynamic", "binary and composition", or "binary only". Nevertheless, when the vector space is constructed by "thermodynamic and composition", "composition only", or "thermodynamic only", there are still more than 20% difference between sensitivity and specificity, even if the dataset is pretty large. Based on these situations, we come to the hypothesis that when there are much difference in record numbers between positive and negative datasets, especially when the dataset is not sufficiently large, the SVM learning machine is inclined to make a biased prediction toward the class with the larger dataset, which results in high false positive or false negative prediction. To validate the hypothesis, we take the following procedure to improve our algorithms:

1. Randomly choose a subset from the larger dataset until the subset has the same number of records as the smaller dataset;

2. Repeat step 1 for ten times to construct ten combinations of this "sub-larger dataset + whole smaller dataset". Make sure that these combinations cover at least 99% of the larger dataset.

3. Training the ten combinations by SVM in the seven vector spaces one by one.

4. Take the average result of the ten combinations as the over all result.

Take Satron's dataset for example, when cut-off value of 0.7 is used, the positive dataset has 141 records while the negative has 420 records. We randomly choose 141 records from the negative dataset for ten times to construct ten subsets. Each of these ten subsets will be trained with the whole positive dataset by SVM in the seven vector spaces. The work of randomly chosen is executed by JAVA program. Only the case when the dataset is Dieter's and cut-off value is 0.6 did not need this randomly chosen scheme. The randomly chosen data of the other five subsets has been supplied in the supplemental material (see additional file 1).

The average results of the randomly process have been supplied in table 6. For Satron's data, the disparity between sensitivity and specificity has been repressed with any of the cut-off values and vector space expanded by any one of the seven attribute combinations. As for Dieter's dataset, the weaken effect on the disparity is not obvious when the dimension of the vector space is high (in this case, the disparity is neglectable), but when the vector space is "thermodynamic and composition", "thermodynamic only" or "composition only" the disparity between sensitivity and specificity is also repressed. These results proved that our strategy to lessen the discrepancy between sensitivity and specificity is workable and can efficiently reduce false positive and false negative during the training processes of machine learning methods.

### The methods are robust for different cut-off values

The cut-off value for siRNA inhibitory activity might be various according to the requests from different experimenter or different experimental intention. Thus we applied three kinds of cut-off values to construct the positive and negative dataset for separate training. For the method of sequence-based statistical model, the prediction results various little under different cases of cut-off values. The accuracy of the sequence-based statistical model is 70.94%, 70.59% and 70.23% for Satron's dataset by cut-off value of 0.7, 0.6 and 0.5 while for Dieter's dataset the corresponding accuracy is 89.35%, 89.47% and 89.43% (table 2). For the method of SVM executed in the space of "binary, thermodynamic and composition", the accuracy in jackknife test on Dieter's dataset is 94.78%, 94.65% and 94.65% for cut-off value of 0.7, 0.6 and 0.5, while the corresponding value on Dieter's dataset is 78.07%, 71.66% and 72.55%, respectively. The little differences from these results show that the three cut-off values affect little on the performance of the two methods we presented in this paper.

### SVM performs better than the sequence-based statistical model

From the results listed in table 2, 3 and 4, we can also see that the SVM model trained in the vector space of "binary, thermodynamic and composition" performs better than the sequence-based model, without discounting the latter (see additional file 2 and 3). From table 5, the high correlation coefficient serves as a strong demonstration of the utility of the sequence-based model and the ability of providing high accuracy than the artificial neural network constructed by Dieter *et al *[16].

## Conclusion

We applied the sequence-based statistical model and support vector machine to the identification of functional siRNA. We constructed three kinds of attribute, namely the binary representation, the thermodynamic profile and the nucleotide composition of a sequence to build the vector space of SVM training machine. Both of the two methods achieved high performance and showed their potential ability to predict efficient siRNAs. We also put forward a procedure to reduce high false positive or false negative values in the situation when the number of records differs greatly between the positive dataset and the negative dataset.

## Methods

### Data set

We chose two datasets for the training of the prediction system. One is Satron's dataset which has been collected from several published experimental reports [8]. The original dataset contains 581 records related with 40 target genes. 8 of the 581 siRNA oligo targeting at least two different genes are deleted from the training dataset. Another four records containing mismatched nucleotides are also deleted. Therefore, the "Satron's dataset" in this research contains 561 records correlated with 40 target genes. Another dataset originated from a recently published high-throughput screening work conducted by Dieter et al [16]. This dataset contains 2431 siRNAs covering 34 target genes. Since the high-throughput dataset is sufficiently large and homogeneous for training, we took the Dieter's dataset as the main assessing dataset. Three cut-off values, 0.5, 0.6 and 0.7, are used to separate the whole dataset into a positive one and a negative one (see additional file 1).

### The sequence-based model [18]

A siRNA oligo nucleotide with 19 or 21 bases can be present as: *R*_1_*R*_2_*R*_3_...*R*_*i*_...*R*_19 _or *R*_1_*R*_2_*R*_3_...*R*_*i*_...*R*_19_*R*_20_*R*_21_, where *R*_*i *_is the nucleotide in the *i*th site. We will take the 19-nt oligos for convenience. All the formulas below will be applicable to the 21-nt oligos. In our research, we suppose that the nucleotide at each site can be treated as an independent element, such that there is no coupling among these sub-sites, then the attribute of the functional and non-functional siRNAs can be formulated, respectively, as:

ψ0+
 MathType@MTEF@5@5@+=feaafiart1ev1aaatCvAUfKttLearuWrP9MDH5MBPbIqV92AaeXatLxBI9gBaebbnrfifHhDYfgasaacH8akY=wiFfYdH8Gipec8Eeeu0xXdbba9frFj0=OqFfea0dXdd9vqai=hGuQ8kuc9pgc9s8qqaq=dirpe0xb9q8qiLsFr0=vr0=vr0dc8meaabaqaciaacaGaaeqabaqabeGadaaakeaaiiGacqWFipqEdaqhaaWcbaacbaGae4hmaadabaaccaGae03kaScaaaaa@307E@(*R*_1_...*R*_5_*R*_6_*R*_7_*R*_8_*R*_9_...*R*_19_) = P1+
 MathType@MTEF@5@5@+=feaafiart1ev1aaatCvAUfKttLearuWrP9MDH5MBPbIqV92AaeXatLxBI9gBaebbnrfifHhDYfgasaacH8akY=wiFfYdH8Gipec8Eeeu0xXdbba9frFj0=OqFfea0dXdd9vqai=hGuQ8kuc9pgc9s8qqaq=dirpe0xb9q8qiLsFr0=vr0=vr0dc8meaabaqaciaacaGaaeqabaqabeGadaaakeaacqWGqbaudaqhaaWcbaGaeGymaedabaGaey4kaScaaaaa@2FD4@ (*R*_1_)...P5+
 MathType@MTEF@5@5@+=feaafiart1ev1aaatCvAUfKttLearuWrP9MDH5MBPbIqV92AaeXatLxBI9gBaebbnrfifHhDYfgasaacH8akY=wiFfYdH8Gipec8Eeeu0xXdbba9frFj0=OqFfea0dXdd9vqai=hGuQ8kuc9pgc9s8qqaq=dirpe0xb9q8qiLsFr0=vr0=vr0dc8meaabaqaciaacaGaaeqabaqabeGadaaakeaacqWGqbaudaqhaaWcbaGaeGynaudabaGaey4kaScaaaaa@2FDC@ (*R*_5_)P6+
 MathType@MTEF@5@5@+=feaafiart1ev1aaatCvAUfKttLearuWrP9MDH5MBPbIqV92AaeXatLxBI9gBaebbnrfifHhDYfgasaacH8akY=wiFfYdH8Gipec8Eeeu0xXdbba9frFj0=OqFfea0dXdd9vqai=hGuQ8kuc9pgc9s8qqaq=dirpe0xb9q8qiLsFr0=vr0=vr0dc8meaabaqaciaacaGaaeqabaqabeGadaaakeaacqWGqbaudaqhaaWcbaGaeGOnaydabaGaey4kaScaaaaa@2FDE@ (*R*_6_)P7+
 MathType@MTEF@5@5@+=feaafiart1ev1aaatCvAUfKttLearuWrP9MDH5MBPbIqV92AaeXatLxBI9gBaebbnrfifHhDYfgasaacH8akY=wiFfYdH8Gipec8Eeeu0xXdbba9frFj0=OqFfea0dXdd9vqai=hGuQ8kuc9pgc9s8qqaq=dirpe0xb9q8qiLsFr0=vr0=vr0dc8meaabaqaciaacaGaaeqabaqabeGadaaakeaacqWGqbaudaqhaaWcbaGaeG4naCdabaGaey4kaScaaaaa@2FE0@ (*R*_7_)P8+
 MathType@MTEF@5@5@+=feaafiart1ev1aaatCvAUfKttLearuWrP9MDH5MBPbIqV92AaeXatLxBI9gBaebbnrfifHhDYfgasaacH8akY=wiFfYdH8Gipec8Eeeu0xXdbba9frFj0=OqFfea0dXdd9vqai=hGuQ8kuc9pgc9s8qqaq=dirpe0xb9q8qiLsFr0=vr0=vr0dc8meaabaqaciaacaGaaeqabaqabeGadaaakeaacqWGqbaudaqhaaWcbaGaeGioaGdabaGaey4kaScaaaaa@2FE2@ (*R*_8_)...P19+
 MathType@MTEF@5@5@+=feaafiart1ev1aaatCvAUfKttLearuWrP9MDH5MBPbIqV92AaeXatLxBI9gBaebbnrfifHhDYfgasaacH8akY=wiFfYdH8Gipec8Eeeu0xXdbba9frFj0=OqFfea0dXdd9vqai=hGuQ8kuc9pgc9s8qqaq=dirpe0xb9q8qiLsFr0=vr0=vr0dc8meaabaqaciaacaGaaeqabaqabeGadaaakeaacqWGqbaudaqhaaWcbaGaeGymaeJaeGyoaKdabaGaey4kaScaaaaa@30D4@ (*R*_19_)

ψ0−
 MathType@MTEF@5@5@+=feaafiart1ev1aaatCvAUfKttLearuWrP9MDH5MBPbIqV92AaeXatLxBI9gBaebbnrfifHhDYfgasaacH8akY=wiFfYdH8Gipec8Eeeu0xXdbba9frFj0=OqFfea0dXdd9vqai=hGuQ8kuc9pgc9s8qqaq=dirpe0xb9q8qiLsFr0=vr0=vr0dc8meaabaqaciaacaGaaeqabaqabeGadaaakeaaiiGacqWFipqEdaqhaaWcbaacbaGae4hmaadabaaccaGae0NeI0caaaaa@3089@ (*R*_1_...*R*_5_*R*_6_*R*_7_*R*_8_*R*_9_...*R*_19_) = P1−
 MathType@MTEF@5@5@+=feaafiart1ev1aaatCvAUfKttLearuWrP9MDH5MBPbIqV92AaeXatLxBI9gBaebbnrfifHhDYfgasaacH8akY=wiFfYdH8Gipec8Eeeu0xXdbba9frFj0=OqFfea0dXdd9vqai=hGuQ8kuc9pgc9s8qqaq=dirpe0xb9q8qiLsFr0=vr0=vr0dc8meaabaqaciaacaGaaeqabaqabeGadaaakeaacqWGqbaudaqhaaWcbaGaeGymaedabaGaeyOeI0caaaaa@2FDF@ (*R*_1_)...P5−
 MathType@MTEF@5@5@+=feaafiart1ev1aaatCvAUfKttLearuWrP9MDH5MBPbIqV92AaeXatLxBI9gBaebbnrfifHhDYfgasaacH8akY=wiFfYdH8Gipec8Eeeu0xXdbba9frFj0=OqFfea0dXdd9vqai=hGuQ8kuc9pgc9s8qqaq=dirpe0xb9q8qiLsFr0=vr0=vr0dc8meaabaqaciaacaGaaeqabaqabeGadaaakeaacqWGqbaudaqhaaWcbaGaeGynaudabaGaeyOeI0caaaaa@2FE7@ (*R*_5_)P6−
 MathType@MTEF@5@5@+=feaafiart1ev1aaatCvAUfKttLearuWrP9MDH5MBPbIqV92AaeXatLxBI9gBaebbnrfifHhDYfgasaacH8akY=wiFfYdH8Gipec8Eeeu0xXdbba9frFj0=OqFfea0dXdd9vqai=hGuQ8kuc9pgc9s8qqaq=dirpe0xb9q8qiLsFr0=vr0=vr0dc8meaabaqaciaacaGaaeqabaqabeGadaaakeaacqWGqbaudaqhaaWcbaGaeGOnaydabaGaeyOeI0caaaaa@2FE9@ (*R*_6_)P7−
 MathType@MTEF@5@5@+=feaafiart1ev1aaatCvAUfKttLearuWrP9MDH5MBPbIqV92AaeXatLxBI9gBaebbnrfifHhDYfgasaacH8akY=wiFfYdH8Gipec8Eeeu0xXdbba9frFj0=OqFfea0dXdd9vqai=hGuQ8kuc9pgc9s8qqaq=dirpe0xb9q8qiLsFr0=vr0=vr0dc8meaabaqaciaacaGaaeqabaqabeGadaaakeaacqWGqbaudaqhaaWcbaGaeG4naCdabaGaeyOeI0caaaaa@2FEB@ (*R*_7_)P8−
 MathType@MTEF@5@5@+=feaafiart1ev1aaatCvAUfKttLearuWrP9MDH5MBPbIqV92AaeXatLxBI9gBaebbnrfifHhDYfgasaacH8akY=wiFfYdH8Gipec8Eeeu0xXdbba9frFj0=OqFfea0dXdd9vqai=hGuQ8kuc9pgc9s8qqaq=dirpe0xb9q8qiLsFr0=vr0=vr0dc8meaabaqaciaacaGaaeqabaqabeGadaaakeaacqWGqbaudaqhaaWcbaGaeGioaGdabaGaeyOeI0caaaaa@2FED@ (*R*_8_)...P19−
 MathType@MTEF@5@5@+=feaafiart1ev1aaatCvAUfKttLearuWrP9MDH5MBPbIqV92AaeXatLxBI9gBaebbnrfifHhDYfgasaacH8akY=wiFfYdH8Gipec8Eeeu0xXdbba9frFj0=OqFfea0dXdd9vqai=hGuQ8kuc9pgc9s8qqaq=dirpe0xb9q8qiLsFr0=vr0=vr0dc8meaabaqaciaacaGaaeqabaqabeGadaaakeaacqWGqbaudaqhaaWcbaGaeGymaeJaeGyoaKdabaGaeyOeI0caaaaa@30DF@ (*R*_19_)     (eq1)

where Pi+
 MathType@MTEF@5@5@+=feaafiart1ev1aaatCvAUfKttLearuWrP9MDH5MBPbIqV92AaeXatLxBI9gBaebbnrfifHhDYfgasaacH8akY=wiFfYdH8Gipec8Eeeu0xXdbba9frFj0=OqFfea0dXdd9vqai=hGuQ8kuc9pgc9s8qqaq=dirpe0xb9q8qiLsFr0=vr0=vr0dc8meaabaqaciaacaGaaeqabaqabeGadaaakeaacqWGqbaudaqhaaWcbaGaemyAaKgabaGaey4kaScaaaaa@303F@(*R*_*i*_) is the probability of nucleotide *R*_*i *_at the sub-site *i *(*i *= 1,2,...,19) for the functional siRNAs, while Pi−
 MathType@MTEF@5@5@+=feaafiart1ev1aaatCvAUfKttLearuWrP9MDH5MBPbIqV92AaeXatLxBI9gBaebbnrfifHhDYfgasaacH8akY=wiFfYdH8Gipec8Eeeu0xXdbba9frFj0=OqFfea0dXdd9vqai=hGuQ8kuc9pgc9s8qqaq=dirpe0xb9q8qiLsFr0=vr0=vr0dc8meaabaqaciaacaGaaeqabaqabeGadaaakeaacqWGqbaudaqhaaWcbaGaemyAaKgabaGaeyOeI0caaaaa@304A@(*R*_*i*_) the corresponding probability for the non-functional siRNAs. The both values of Pi+
 MathType@MTEF@5@5@+=feaafiart1ev1aaatCvAUfKttLearuWrP9MDH5MBPbIqV92AaeXatLxBI9gBaebbnrfifHhDYfgasaacH8akY=wiFfYdH8Gipec8Eeeu0xXdbba9frFj0=OqFfea0dXdd9vqai=hGuQ8kuc9pgc9s8qqaq=dirpe0xb9q8qiLsFr0=vr0=vr0dc8meaabaqaciaacaGaaeqabaqabeGadaaakeaacqWGqbaudaqhaaWcbaGaemyAaKgabaGaey4kaScaaaaa@303F@(*R*_*i*_) and Pi−
 MathType@MTEF@5@5@+=feaafiart1ev1aaatCvAUfKttLearuWrP9MDH5MBPbIqV92AaeXatLxBI9gBaebbnrfifHhDYfgasaacH8akY=wiFfYdH8Gipec8Eeeu0xXdbba9frFj0=OqFfea0dXdd9vqai=hGuQ8kuc9pgc9s8qqaq=dirpe0xb9q8qiLsFr0=vr0=vr0dc8meaabaqaciaacaGaaeqabaqabeGadaaakeaacqWGqbaudaqhaaWcbaGaemyAaKgabaGaeyOeI0caaaaa@304A@(*R*_*i*_) can be derived from a positive and negative training datasets, respectively. The superscript "+" or "-" of *ψ *indicates the attribute quality of the dataset as positive or negative, respectively. The subscript 0 of *ψ *indicates that the attribute function is formed by independent probabilities in which there was no coupling effect among subsites. Based on this approach, prediction can be performed: if *ψ*^+ ^> *ψ*^-^, then the target siRNA is deemed to be a functional siRNA; if *ψ*^+ ^<*ψ*^-^, then a non-functional one. We constructed a discriminant function given by:

Δ(*R*_1_...*R*_5_*R*_6_*R*_7_*R*_8_*R*_9_...*R*_19_) = *ω*^+^ψ0+
 MathType@MTEF@5@5@+=feaafiart1ev1aaatCvAUfKttLearuWrP9MDH5MBPbIqV92AaeXatLxBI9gBaebbnrfifHhDYfgasaacH8akY=wiFfYdH8Gipec8Eeeu0xXdbba9frFj0=OqFfea0dXdd9vqai=hGuQ8kuc9pgc9s8qqaq=dirpe0xb9q8qiLsFr0=vr0=vr0dc8meaabaqaciaacaGaaeqabaqabeGadaaakeaaiiGacqWFipqEdaqhaaWcbaacbaGae4hmaadabaaccaGae03kaScaaaaa@307E@(*R*_1_...*R*_5_*R*_6_*R*_7_*R*_8_*R*_9_...*R*_19_) - *ω*^-^ψ0−
 MathType@MTEF@5@5@+=feaafiart1ev1aaatCvAUfKttLearuWrP9MDH5MBPbIqV92AaeXatLxBI9gBaebbnrfifHhDYfgasaacH8akY=wiFfYdH8Gipec8Eeeu0xXdbba9frFj0=OqFfea0dXdd9vqai=hGuQ8kuc9pgc9s8qqaq=dirpe0xb9q8qiLsFr0=vr0=vr0dc8meaabaqaciaacaGaaeqabaqabeGadaaakeaaiiGacqWFipqEdaqhaaWcbaacbaGae4hmaadabaaccaGae0NeI0caaaaa@3089@(*R*_1_...*R*_5_*R*_6_*R*_7_*R*_8_*R*_9_...*R*_19_)     (eq2)

where *ω*^+ ^and *ω*^- ^are the weight factors for the attribute functions derived from the positive and negative training dataset, respectively. Except in special cases, these values are generally set to 1. (*ω*^+ ^= *ω*^- ^= 1). Thus, the criterion of a functional siRNA prediction for a given RNA oligo can be formulated as follows: The RNA oligo is functional if Δ > 0, and it is non-functional if Δ ≤ 0.

Generally speaking, if the coupling effects of the *μ *(*μ *= 1,2,3...) of the closet neighboring nucleotide need to be considered, then eq. 1 should be modified according to the *μ *th-order Markov chain theory and the attribute function *ψ*_0 _should be replaced by *ψ*_*μ*_. In this research, we do not yet consider the coupling effects.

#### Support Vector Machine

The support vector machine has been widely used in many areas for its attractive features. It is a kind of machine learning methodology based on statistical learning theory. Briefly speaking, the SVM defines a feature space (often with a higher dimension) and map the input vectors into this space. In the feature space, SVM tries to classify the data points into two classes by seeking an optimized classifier called hyperplane. It has also been applied in the field of multiple classification problems recently. In this paper, we use it to classify functional siRNAs from non-functional siRNAs. To learn more about the mechanism of SVM, readers can consult a series of previous studies for further explanation on the procedure of how to use the support vector machine [10,12-14,19,20].

To construct the vector space, we introduced three kinds of attributes, representing three aspects of a siRNA oligo. The first one is the binary system by which the 5 nucleotides of *a*s, *t*s, *u*s, *c*s and *g*s are coded by 4-D vectors composed of only 0 and 1 (adenine = 0001, uracil or thymine = 0010, cytosine = 0100, guanine = 1000). Then a 19-nt siRNA is represented by a 76-dimension vector in the SVM input or a 21-nt siRNA can be represented by an 84-dimension vector. The second attribute describes the thermodynamic profile of the siRNAs. The nearest neighbour model was introduced to calculate the pentamer sub-sequences of the siRNA oligos under examination to reflect its internal stability values [21]. For the terminal four bases of the 3'end of the oligos, the targeting sequences would be extended for calculation. If extension is impossible (for example when the targeting sites on the targeted gene is before the third base of the gene), the thermodynamic value of the terminal four bases are set to be zero. The third attribute defines the composition features of siRNA sequences including 4 single nucleotide and 16 di-nucleotide compositions.

The parameters for the training process by SVM were set to be default values. The software used to implement SVM was SVM_light by Joachims [22]. The training process was carried out on the computer we used (Dell OptiPlex GX270 computer with an Intel Pentium4 2.80 GHz CPU).

To objectively assess our prediction system, we employed the following measurements for sensitivity, specificity, accuracy and receiver operating characteristics (ROC):







*ROC *(*receiver operating characteristics*)



TP, true positive; TN: true negative; FP, false positive; FN, false negative.

## Authors' contributions

Peilin Jia conceived of the study, carried out the algorithm design and realization and helped to draft the manuscript. Yudong Cai and Yixue Li conceived of the study, helped to improve the algorithm and to draft the manuscript. Tieliu Shi, Yixue Li and Yudong Cai helped to revise the manuscript.

## Supplementary Material

Additional File 2 Results by the method of SeqSta. Click here for file

Additional File 3 Results by the method of SVM. All results, including those tested on the whole dataset and those gotten by the ten randomly chosen processes, are listed in this file. Click here for file

Additional File 4 Comparison results between our methods and Dieter's method. Click here for file

Additional File 1 The two datasets we used are described in this file, including all the sequences of the siRNAs and the density distribution of inhibitory activities of each dataset. We also described which siRNAs are selected during the ten random processes under different cut-off values in this file. Click here for file

## Figures and Tables

**Table 1:** Three cut-off values have been used to generate positive and negative subsets from the Dieter's and Satron's datasets respectively. Six columns indicating six combinations of positive and negative subsets are listed with the number of siRNAs in each subset.

	Dieter's Dataset			Satron's Dataset		
	
	Cut-off 0.5	Cut-off 0.6	Cut-off 0.7	Cut-off 0.5	Cut-off 0.6	Cut-off 0.7
Number of siRNAs in the positive dataset	1585	1180	734	221	178	141
Number of siRNAs in the negative dataset	846	1251	1697	340	383	420
All	2431	2431	2431	561	561	561

**Table 2:** The self-consistency and jackknife results for the sequence-based method trained by the six combinations listed in table 1.

	Dieter's Dataset		Satron's Dataset	
	Self-Consistency	Jackknife	Self-consistency	Jackknife
Cut-Off 0.7				
Accuracy	89.88%	89.35%	76.83%	70.94%
Sensitivity	94.28%	93.87%	75.18%	68.09%
Specificity	87.98%	87.39%	77.38%	71.90%
Pearson	0.658	0.6594	0.4816	0.4021
ROC	0.975	0.9698	0.8333	0.7557

Cut-Off 0.6				
Accuracy	90.79%	89.47%	75.04%	70.59%
Sensitivity	91.27%	89.49%	74.72%	67.42%
Specificity	90.33%	89.45%	75.20%	72.06%
Pearson	0.8298	0.8264	0.4699	0.3944
ROC	0.9735	0.9686	0.8169	0.7381

Cut-Off 0.5				
Accuracy	90.25%	89.43%	77.18%	70.23%
Sensitivity	88.33%	87.70%	75.57%	68.78%
Specificity	93.85%	92.67%	78.24%	71.18%
Pearson	0.8288	0.8278	0.4446	0.3851
ROC	0.9751	0.97	0.8353	0.7675

**Table 3:** The jackknife results for the method of support vector machine trained by the six combinations listed in table 1. Three attributes have been defined, namely binary system (denoted by "A" in the table), thermodynamic profile ("B" in the table) and composition ("C" in the table). Seven combinations of the attributes are put forward, which are A+B+C (means "binary, thermodynamic and composition"), A+B (means "binary and thermodynamic"), B+C (means "thermodynamic and composition"), A+C (means "thermodynamic and composition"), A (means "binary only"), B (means "thermodynamic only") and C (means "composition"). The self-consistency and jackknife test are executed in all the seven vector space respectively to compare the contribution from each of the three attributes. To save space, here we just listed the results of jackknife test. This table lists results of Dieter's dataset. See table 4 for Satron's dataset. Self-consistency results have been placed in the supplemental file (see additional file 3) Dieter's dataset, jackknife test:

	A+B+C	A+B	B+C	A+C	A	B	C
Cut-off 0.7							
Accuracy	94.78%	94.90%	85.97%	94.86%	**96.13%**	78.69%	81.65%
Sensitivity	86.51%	87.87%	67.98%	87.19%	91.14%	48.64%	57.90%
Specificity	98.35%	97.94%	93.75%	98.17%	98.29%	91.69%	91.93%
Pearson	0.9726	0.9752	0.8522	0.9749	0.9808	0.7189	0.7377
ROC	0.9899	0.9922	0.9302	0.9913	0.9952	0.8411	0.8809

Cut-off 0.6							
Accuracy	94.65%	96.01%	83.83%	95.80%	**96.71%**	76.31%	80.09%
Sensitivity	94.32%	96.19%	82.37%	95.42%	96.61%	73.39%	79.66%
Specificity	94.96%	95.84%	85.21%	96.16%	96.80%	79.06%	80.50%
Pearson	0.9735	0.9786	0.8469	0.9775	0.9825	0.7181	0.7619
ROC	0.9912	0.9947	0.9223	0.9937	0.9967	0.8436	0.885

Cut-off 0.5							
Accuracy	94.65%	95.56%	83.67%	95.23%	**96.42%**	77.46%	79.47%
Sensitivity	96.53%	97.10%	90.85%	96.85%	97.79%	88.71%	86.75%
Specificity	91.13%	92.67%	70.21%	92.20%	93.85%	56.38%	65.84%
Pearson	0.9726	0.974	0.8415	0.9761	0.98	0.7172	0.741
ROC	0.9906	0.9928	0.9121	0.9926	0.9951	0.8435	0.8668

**Table 4:** Everything is the same with table 3 except that the dataset is from Satron's work. Satron's Dataset, Jackknife test:

	A+B+C	A+B	B+C	A+C	A	B	C
Cut-off 0.7							
Accuracy	78.07%	74.87%	75.22%	**78.43%**	76.47%	74.87%	74.87%
Sensitivity	21.99%	9.93%	4.26%	24.11%	16.31%	0.00%	0.00%
Specificity	96.09%	96.67%	99.05%	96.97%	96.67%	100.00%	100.00%
Pearson	0.4369	0.4458	0.4032	0.4562	0.4432	0.2855	0.4013
ROC	0.7476	0.7488	0.7309	0.7648	0.755	0.6554	0.7381

Cut-off 0.6							
Accuracy	71.66%	68.98%	**73.26%**	73.08%	70.23%	68.27%	70.41%
Sensitivity	31.46%	24.72%	26.97%	34.27%	29.78%	0.00%	14.61%
Specificity	90.34%	89.56%	94.78%	91.12%	89.03%	100.00%	96.34%
Pearson	0.4465	0.4327	0.4254	0.4477	0.4533	0.3273	0.3698
ROC	0.7363	0.7228	0.7293	0.7414	0.7375	0.6679	0.7029

Cut-off 0.5							
Accuracy	**72.55%**	71.12%	69.34%	72.19%	70.77%	63.99%	68.09%
Sensitivity	58.37%	54.75%	47.96%	56.56%	55.66%	40.27%	34.39%
Specificity	81.76%	81.76%	83.24%	82.35%	80.59%	79.41%	90.00%
Pearson	0.4868	0.4814	0.4642	0.4976	0.4597	0.3625	0.3994
ROC	0.7706	0.7721	0.7508	0.7846	0.755	0.685	0.7132

**Table 5:** Compare with Dieter's results. Using the same training and testing dataset, both of the two methods have been applied to compute the pearson correlation coefficient. Also, cut off value should be specified as 0.5, 0.6 or 0.7. Here we just showed the result when cut off value is 0.6. The results when cut-off value is 0.5 or 0.7 are detailed in supplemental file (see additional file 4), which also shows the accuracy, sensitivity, specificity and ROC for the two methods. For more info about Dieter's work or the explanation about the datasets used by them, please consult [15]. SVM, cut-off 0.6

Pearson	All(249)	All human(198)	hE2(139)	Rodent(51)
All(2182)	0.9771	0.9769	0.9743	0.9713
All human(1744)	0.9721	0.9722	0.9689	0.9639
Human E2s(1229)	0.9653	0.9644	0.9606	0.9593
Rodent(438)	0.9057	0.9077	0.895	0.8806
Random all (1091)	0.9660	0.9673	0.9651	0.9510
Random all (727)	0.9343	0.9369	0.9387	0.9125
Random all (545)	0.9249	0.9252	0.9206	0.9154
Random all (218)	0.8502	0.8645	0.8570	0.7713
All-19	0.9436			
All human-19	0.9387			
Rodent-19	0.8487			

SeqSta, cut-off 0.6				

Pearson	All(249)	All human(198)	hE2(139)	Rodent(51)

All(2182)	0.8562	0.8557	0.8452	0.8520
All human(1744)	0.8104	0.8106	0.8007	0.8008
Human E2s(1229)	0.7294	0.7294	0.7353	0.7257
Rodent(438)	0.7761	0.7688	0.7608	0.7912
Random all(1091)	0.8632	0.8619	0.8472	0.8644
Random all(727)	0.7953	0.8023	0.7830	0.7523
Random all(545)	0.7812	0.7785	0.7679	0.7748
Random all(218)	0.7017	0.6941	0.6681	0.7292
All-19	0.8224			
All human-19	0.7809			
Rodent-19	0.7097			

**Table 6:** By randomly choosing 10 subsets from each of the 5 datasets whose positive subset departs from the negative one greatly, we try to alleviate the bias between the value of sensitivity and specificity. Here we show the average results tested on Satron's dataset with cut-off value as 0.6. All the sub-datasets and results have been supplied in the additional file (see additional file 1 and additional file 3).

	A+B+C	A+B	B+C	A+C	A	B	C
Accuracy	68.43 ± 1.45%	67.13 ± 1.95%	65.37 ± 2.19%	68.09 ± 2.14%	68.76 ± 2.59%	61.55 ± 2.56%	63.90 ± 2.52%
Sensitivity	69.05 ± 1.20%	69.78 ± 2.98%	64.33 ± 3.41%	67.25 ± 3.19%	69.72 ± 3.63%	63.93 ± 2.96%	58.03 ± 3.63%
Specificity	67.81 ± 3.07%	64.49 ± 2.21%	66.4 ± 3.36%	68.93 ± 2.53%	67.81 ± 2.68%	59.16 ± 3.63%	69.78 ± 4.32%
Pearson	0.4683 ± 0.03219	0.4618 ± 0.03205	0.4112 ± 0.03306	0.4447 ± 0.03097	0.4718 ± 0.03558	0.3758 ± 0.02486	0.3196 ± 0.02920
ROC	0.7452 ± 0.02383	0.7255 ± 0.02886	0.7093 ± 0.02450	0.7367 ± 0.02454	0.7411 ± 0.02933	0.6768 ± 0.02220	0.6715 ± 0.02659

Satron's dataset, cut-off value = 0.6. Record in the positive part before randomly chosen: 178; Records in the negative part before randomly chosen: 383. Each of the 10 randomly chosen subsets has 178 records as positive and 178 out of 383 as negative part. The pseudorandom numbers were generated by the java class of java.lang.Random.

## References

[B1] Mello CC, Jr DC (2004). Revealing the world of RNA interference. Nature.

[B2] Hannon GJ, Rossi JJ (2004). Unlocking the potential of the human genome with RNA interference. Nature.

[B3] Wang L, Mu FY (2004). A Web-based design center for vector-based siRNA and siRNA cassette. Bioinformatics.

[B4] Reynolds A, Leake D, Boese Q, Scaringe S, Marshall WS, khvorova A (2004). Rational siRNA design for RNA interference. Nature Biotechnology.

[B5] Ui-Tei K, Naito Y, Takahashi F, Haraguchi T, Ohki-Hamazaki H, Juni A, Ueda R, Saigo K (2004). Guidelines for the selection of highly effective siRNA sequences for mammalian and chick RNA interference. Nucl Acids Res.

[B6] Henschel A, Buchholz F, Habermann B (2004). DEQOR: a web-based tool for the design and quality control of siRNAs. Nucl Acids Res.

[B7] Satrom P, Snove JO (2004). A comparison of siRNA efficacy predictors. Biochemical and Biophysical Research Communications.

[B8] Saetrom P (2004). Predicting the efficacy of short oligonucleotides in antisense and RNAi experiments with boosted genetic programming. Bioinformatics.

[B9] Chou KC (2001). Using subsite coupling to predict signal peptides. Protein Engineering.

[B10] Vapnik (1995). The Nature of Statistical Learning Theory. Springer.

[B11] Brown MPS, Grundy WN, Lin D, Cristianini N, Sugnet CW, Furey TS, Ares M, Haussler D, Jr. (2000). Knowledge-based analysis of microarray gene expression data by using support vector machines. PNAS.

[B12] Cai YD, Liu XJ, Xu X, Zhou GP (2001). Support Vector Machines for predicting protein structural class. BMC Bioinformatics.

[B13] Cai YD, Lin S, Chou KC (2003). Support vector machines for prediction of protein signal sequences and their cleavage sites. Peptides.

[B14] Cai YD, Liu XJ, Xu XB, Chou KC (2000). Support Vector Machines for Prediction of Protein Subcellular Location. Molecular Cell Biology Research Communication.

[B15] Huesken D, Lange J, Mickanin C, Weiler J, Asselbergs F, Warner J, Meloon B, Engel S, Rosenberg A, Cohen D, Labow M, Reinhardt M, Natt F, Hall J (2005). Design of a genome-wide siRNA library using an artificial neural network. Nat Biotech.

[B16] Khvorova A, Reynolds A, Jayasena SD (2003). Functional siRNAs and miRNAs Exhibit Strand Bias. Cell.

[B17] Chou KC (2001). Predicting of Protein Signal Sequences and Their Cleavage Sites.. PROTEINS: Structure, Function, and Genetics.

[B18] Cai YD, Liu XJ, Xu X, Chou KC (2001). Support vector machines for predicting HIV protease cleavage sites in protein. Journal of Computational Chemistry.

[B19] Hua S, Sun Z (2001). Support vector machine approach for protein subcellular localization prediction. Bioinformatics.

[B20] M.FREIER SUSAN, KIERZEK RYSZARD, A.JAEGER JOHN, SUGIMOTO NAOKI, H.CARUTHERS MARVIN, NEILSON THOMAS, H.TURNER DOUGLAS (1986). Improved free-eneregy parameters for predictions of RNA duplex stability. PNAS.

